# Thymol Regulates the Expression of Antioxidant-Related Messenger RNAs and Improves Bovine Secondary Follicles Development and Antrum Formation In Vitro

**DOI:** 10.3390/ijms27219451

**Published:** 2026-10-23

**Authors:** Danisvânia Ripardo do Nascimento, Pedro Alves Aguiar Barroso, Venância Nunes Azevedo, Ernando Igo Teixeira de Assis, Solano Dantas Martins, Valdevane Rocha Araújo, Geovany Amorim Gomes, Mariana Aragão Matos Donato, Christina Alves Peixoto, José Roberto Viana Silva

**Affiliations:** 1 Laboratory of Biotechnology and Physiology of Reproduction, Federal University of Ceara, Sobral 62041-040, CE, Brazil; danisvania@alu.ufc (D.R.d.N.); pedro.alves@faculdadeviasapiens.com.br (P.A.A.B.); venanciaazevedo@alu.ufc.br (V.N.A.); igo.texeira@flucianofeijao.com.br (E.I.T.d.A.); 2 Laboratory of Research in Reproductive Physiology, Parnaíba Delta Federal University, Parnaíba 64202-020, PI, Brazil; solanomartins@alu.ufc.br (S.D.M.); valdevane.araujo@ufdpar.edu.br (V.R.A.); 3 Laboratory of Organic Chemistry, State University of Acarau Valley, Sobral 62040-370, CE, Brazil; geovany_amorim@uvanet.br; 4 Laboratory of Ultrastructure, CPqAM/FIOCRUZ, Federal University of Pernambuco, Recife 50670-901, PE, Brazil; mariana.donato@ufpe.br (M.A.M.D.); christina.peixoto@fiocruz.br (C.A.P.)

**Keywords:** oxidative stress, granulosa cell viability, redox homeostasis

## Abstract

This study investigated the effects of 1, 10, or 100 μg/mL thymol on bovine secondary follicles cultured in vitro for 18 days at 38.5 °C with 5% CO_2_. After culture, the following parameters were evaluated: (1) the follicular growth, antrum formation, viability, and ultrastructure, (2) the levels of malondialdehyde, reduced glutathione, and nitrite in the culture medium, and (3) mRNA levels of catalase (CAT), glutathione peroxidase 1 (GPX1), superoxide dismutase (SOD), peroxiredoxin 6 (PRDX6), proliferating cell nuclear antigen (PCNA), and growth differentiation factor 9 (GDF-9). The results showed that 10 μg/mL thymol increased cell viability, antrum formation and follicular development. At day 18, nitrite levels were higher in medium of follicles cultured with 10 μg/mL thymol than those found in control medium alone or with 1 μg/mL thymol. Gene expression analysis revealed that 10 μg/mL thymol increased catalase and glutathione peroxidase 1 mRNA levels, while the expression of other transcripts was unaffected. Transmission electron microscopy showed that granulosa cells exhibited rounded nuclei, elongated mitochondria, and sparse cytoplasmic vacuoles in all treatments, except for those cultured in control medium that had signs of degeneration. In conclusion, 10 μg/mL thymol increased mRNA expression of catalase and glutathione peroxidase 1, stimulated antrum and preserved granulosa cells of bovine secondary follicles in vitro.

## 1. Introduction

The in vitro culture of early ovarian follicles has received significant attention due to its potential to improve reproductive outcomes in cattle and promote a better understanding of the process of folliculogenesis [1,2,3]. The success of this biotechnology largely depends on the culture conditions, particularly the composition of culture medium. The in vitro follicular development can be monitored through markers of cellular activity, such as PCNA, that is a cofactor of DNA polymerase and it is widely used as an indicator of granulosa cell proliferation [4]. Additionally, GDF-9, an oocyte-secreted paracrine factor, reflects oocyte developmental competence and oocyte-granulosa cell communication during folliculogenesis [5,6]. Maintaining cellular homeostasis and protection against oxidative stress have emerged as critical aspects of in vitro follicle culture [1,2,7,8]. The cellular enzymatic system plays a central role in protecting granulosa cells and oocytes from oxidative damage during in vitro follicle growth. The SOD catalyzes the dismutation of the superoxide anion into hydrogen peroxide and molecular oxygen, while CAT and GPX1 subsequently convert hydrogen peroxide into water, preventing the accumulation of reactive oxygen species [9,10]. The PRDX6 provides an additional layer of protection by reducing hydrogen peroxide and repairing oxidized membrane phospholipids, and its expression has been shown to respond to phenolic compounds such as thymol in cultured bovine ovarian tissue [11]. Some studies have shown that when cellular defenses are unable to control oxidative stress, reductions in cell viability, oocyte quality, and embryonic development are commonly observed [9,12]. Therefore, the presence of antioxidants in culture medium is essential for mitigating oxidative damage and enhancing the effectiveness of in vitro culture systems.

Thymol (2-isopropyl-5-methylphenol), a natural monoterpene phenolic compound found in thyme oil, is known for its potent antioxidant activity in various biological systems. Its structure has a phenolic hydroxyl group, which confers antioxidant properties that can act directly in the neutralization of free radicals. It can also promote an increase in the activity of both enzymatic and non-enzymatic endogenous antioxidant systems, since it acts in the chelation of metal ions [13,14,15,16]. Recent studies have shown that thymol protects ovarian cells from oxidative stress, thus increasing cell viability and function [11]. The levels of malondialdehyde (MDA), reduced glutathione (GSH) and nitric oxide have been used to indicate cellular activity. The presence of high MDA levels is associated with increased oxidative stress, particularly lipid peroxidation [17]. Nitric oxide is a signaling molecule involved in the regulation of cellular functions [18,19]. The reduced GSH levels are key indicators of antioxidant protection and preservation of redox homeostasis [20,21,22]. Thus, it is hypothesized that the presence of thymol in the culture medium of bovine secondary follicles improve the in vitro environment for follicular development by increasing follicular diameter and antrum formation, enhancing cell viability, and reducing oxidative stress induced damage.

This study aims to investigate the effects of thymol on growth, antrum formation, viability, ultrastructure, the expression of mRNA of CAT, GPX1, SOD, PRDX6, PCNA, and GDF-9 of bovine secondary follicles cultured in vitro for 18 days, as well as on levels of reduced GSH, nitrite, and MDA in the culture medium, and on the mRNA expression of CAT, GPX1, SOD, PRDX6, PCNA, and GDF-9.

## 2. Results

### 2.1. Follicular Growth and Viability During In Vitro Culture

The supplementation of culture medium with 10 or 100 µg/mL thymol increased the rate of antrum formation (*p* < 0.05). Notably, 10 µg/mL thymol also increased the percentage of growing follicles at the end of the culture period (*p* < 0.05). No significant effect of thymol on the percentage of normal follicles was observed (*p* > 0.05, Table 1).

**Figure 1:**
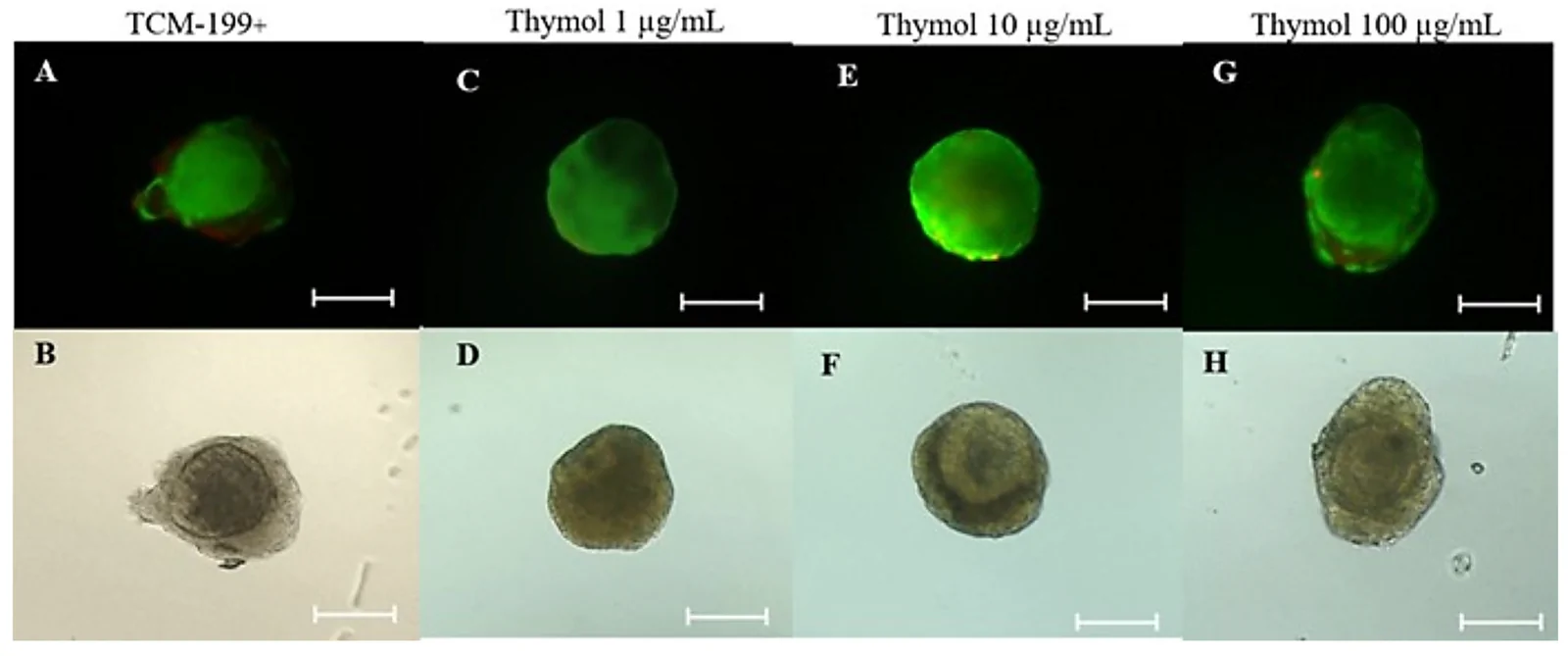
Secondary follicles stained with calcein-AM (green) and ethidium homodimer (red) after 18 days of culture in TCM-199^+^ alone (**A**,**B**) or with 1 (**C**,**D**), 10 (**E**,**F**) or 100 µg/mL (**G**,**H**) thymol. Scale bars represent 200 µm. n = 30 follicles per treatment.

**Figure 2:**
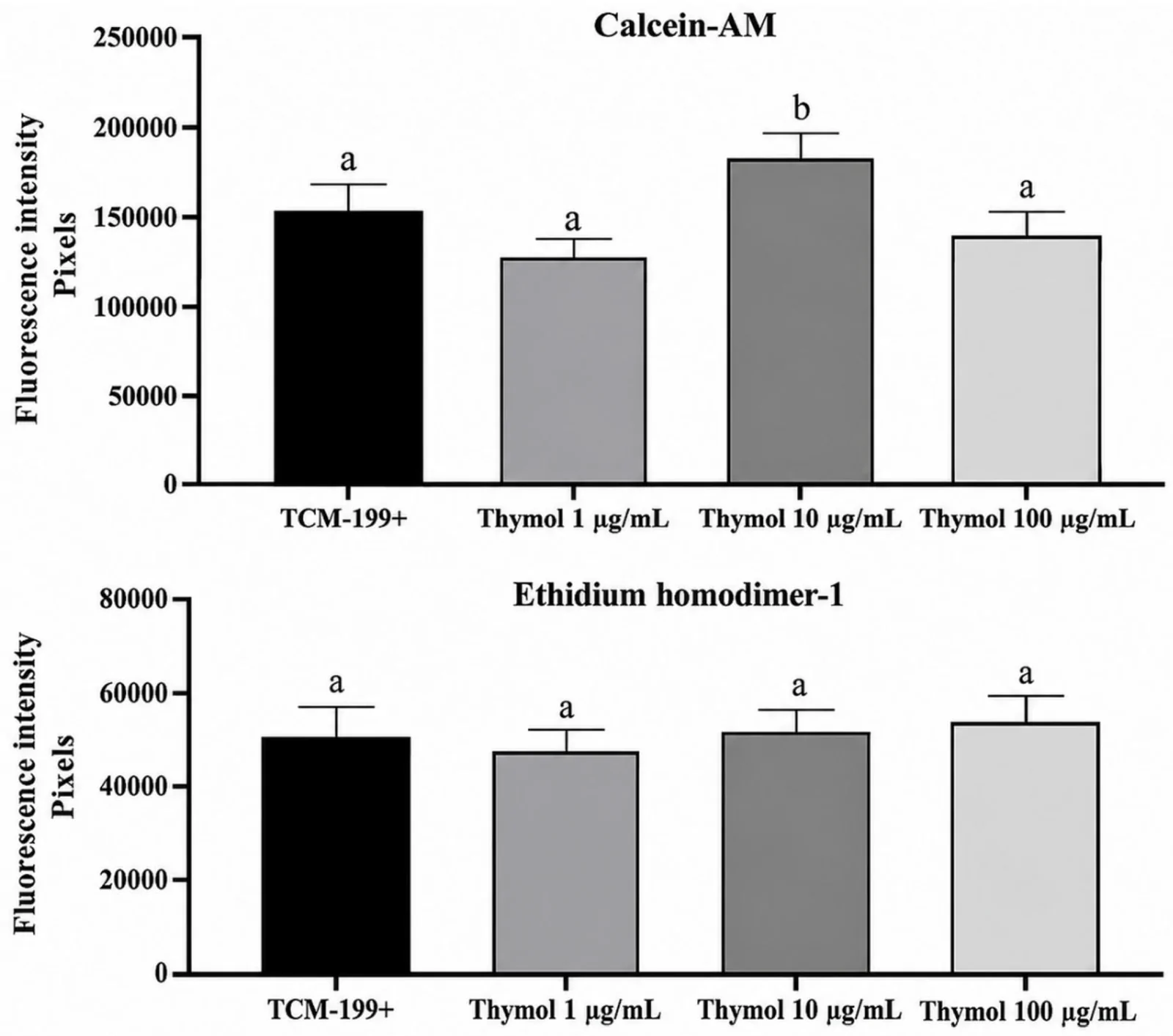
Fluorescence intensity of calcein-AM and ethidium homodimer-1 in bovine secondary follicles after 18 days of culture in TCM-199^+^ alone or with 1, 10 or 100 µg/mL thymol. a, b represent statistically significant differences between treatments (*p* < 0.05). n = 30 follicles per treatment.

In relation to viability markers (Figure 1), a higher fluorescence intensity of calcein-AM was observed in follicles cultured with 10 µg/mL thymol, compared to follicles from other treatments (*p* < 0.05, Figure 2). No differences were seen in the fluorescence intensity of ethidium homodimer-1 between treatments (*p* > 0.05, Figure 2). Follicles cultured in all treatments had an increase in follicular diameter from day 0 to day 18 (*p* < 0.05); however, no differences were found in follicular growth rates among the treatments (*p* > 0.05, Table 2).

### 2.2. Ultrastructural Characteristics of Cultured Secondary Follicles

**Figure 3:**
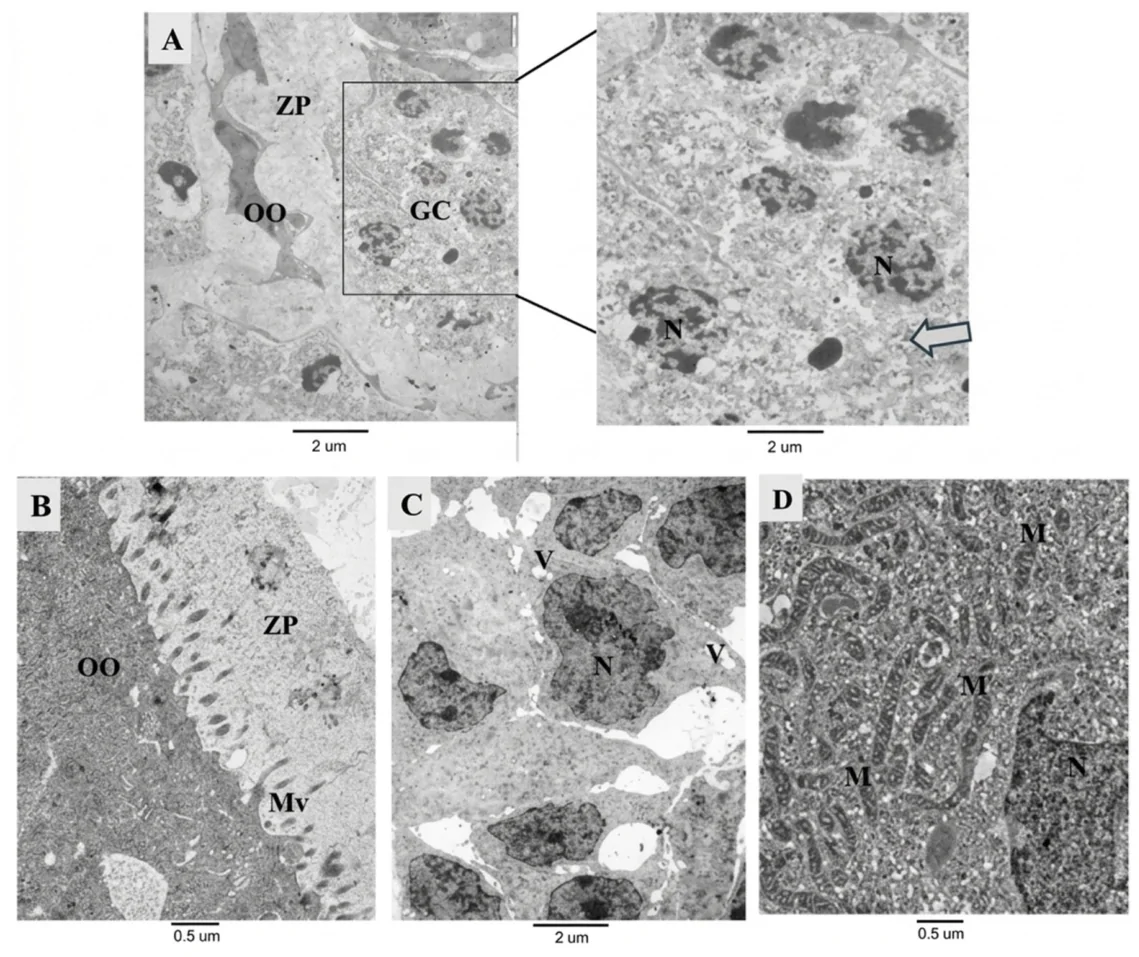
Ultrastructural characteristics of the oocyte and granulosa cells of bovine secondary follicles after 18 days of in vitro culture in TCM-199^+^ alone (**A**) or supplemented with 1 (**B**), 10 (**C**), and 100 µg/mL (**D**) thymol. Oocyte (OO), zona pellucida (ZP), oocyte microvilli (Mv) nuclei (N), mitochondria (M), and vacuoles (V). The arrow indicates cytoplasm of granulosa cells with unrecognizable organelles. n = 8 follicle per treatment. Micrographs are representative of qualitative ultrastructural observations.

Follicles cultured in control medium exhibited oocytes with poorly defined cytoplasmic content and an irregularly shaped zona pellucida, as well as granulosa cells with fragmented chromatin and indistinguishable organelles. This indicates that 18 days of culture causes considerable ultrastructural damage, corroborating data described in the literature (Figure 3A). Oocytes from follicles cultured with thymol, at the various concentrations tested, displayed a continuous ooplasm delimited by an intact zona pellucida, into which numerous oocyte microvilli (Mv) projected (Figure 3B). Follicular granulosa cells cultured in medium enriched with thymol appeared well-interconnected and exhibited rounded nuclei with preserved chromatin (Figure 3C), as well as cytoplasm containing intact, elongated mitochondria (Figure 3D). It is worth noting that the presence of thymol in the culture medium did not cause significant deleterious ultrastructural effects compared to follicles cultured in TCM alone.

### 2.3. Effects of Thymol on Levels of MDA, GSH and Nitrite

**Figure 4:**
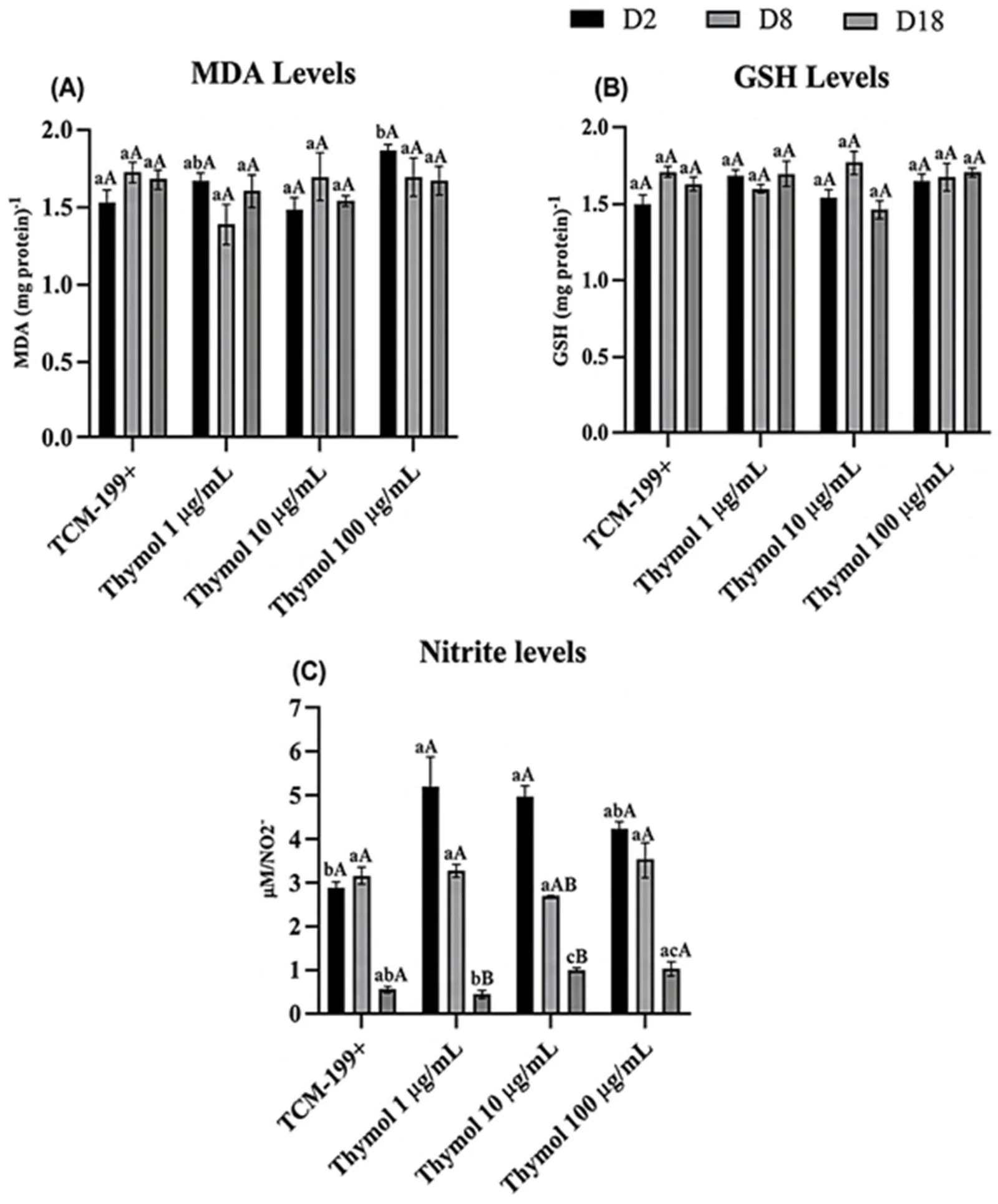
Levels (mean ± SEM) of malondialdehyde (MDA) (**A**), glutathione (GSH) (**B**) and nitrite (NO^2−^) (**C**) in the culture medium at day 2 (D2), day 8 (D8), and day 18 (D18). A, B: Represent statistically significant differences in the same treatment on different days (*p* < 0.05). a, b: Represent statistically significant differences in the same day in different treatments (*p* < 0.05).

After two days, the MDA levels in the medium of follicles cultured with 1 or 10 µg/mL thymol were similar to the control, but the presence of 100 µg/mL thymol resulted in an increase in MDA when compared to those seen in control medium alone or with 10 µg/mL thymol (*p* < 0.05, Figure 4A). No effect of thymol on MDA levels was seen after 8 and 18 days (*p* > 0.05). Additionally, no significant differences in MDA levels were found when comparing treatments across different culture periods (*p* > 0.05, Figure 4A).

Neither thymol nor the culture period influenced GSH levels (Figure 4B). However, after 2 days of culture, higher levels of nitrite were observed in the medium of follicles cultured with 1 and 10 µg/mL thymol compared to the control group (*p* < 0.05). No significant differences were observed between the treatments on day 8 (*p* > 0.05). After 18 days, follicles cultured with 10 µg/mL thymol showed higher nitrite production compared to the other treatments (*p* < 0.05).

### 2.4. Effects of Thymol on mRNA Expression

**Figure 5:**
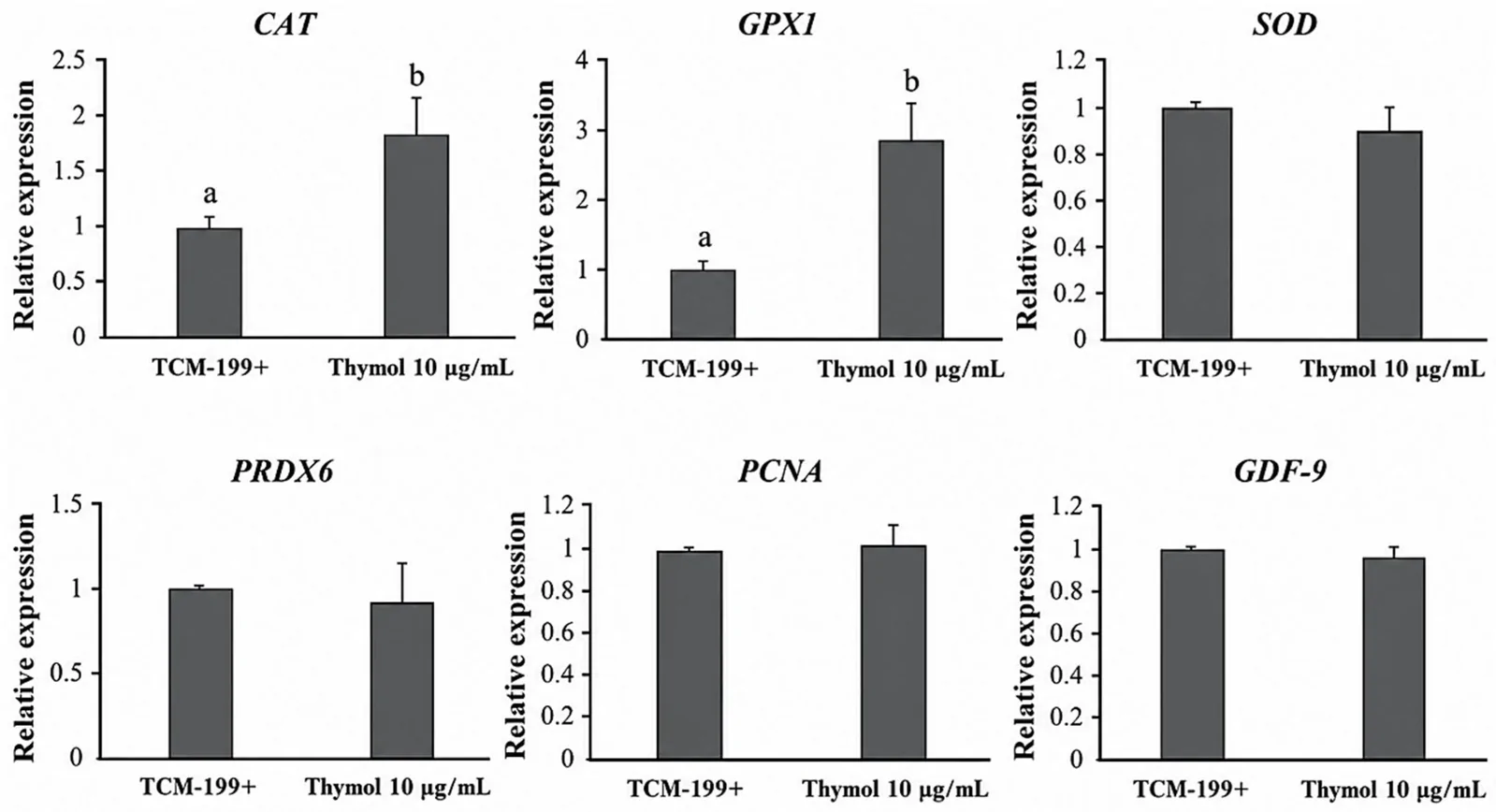
Relative Expression of mRNA for CAT, GPX1, SOD, PRDX6, PCNA and GDF-9 in bovine secondary follicles cultured with 10 µg/mL thymol for 18 days. n = 64 follicles per experimental group.

After 18 days of in vitro culture, follicles cultured in the presence of 10 μg/mL thymol showed higher levels of mRNA for CAT and GPX1 when compared to those cultured in control medium (*p* < 0.05, Figure 5). In contrast, no differences were detected between treatments in the relative mRNA levels of SOD, PRDX6, PCNA, and GDF-9 (*p* > 0.05) (Figure 5).

## 3. Discussion

This study demonstrated that thymol increased the rate of antrum formation and the percentage of follicles that remained in active development after 18 days of in vitro culture. Follicular growth depends on the metabolic activity of granulosa cells and their capacity to sustain the progressive formation of the antral cavity, two processes that are sensitive to the redox conditions maintained throughout the culture period [2,23]. Caetano Filho et al. [11] demonstrated that thymol preserved stromal cell integrity, promoted primordial follicle activation, and maintained collagen fiber structure in bovine ovarian tissue cultured in vitro, indicating that this compound creates favorable conditions for cellular activity in ovarian tissue.

In the present study, follicles cultured with 10 µg/mL thymol had increased antrum formation, which results from a coordinated secretory and osmotic process driven by the metabolic activity of granulosa cells [24,25]. These cells produce and secrete proteoglycans and glycosaminoglycans, including hyaluronic acid and perlecan, which accumulate in the intercellular space, generate an osmotic gradient, and promote fluid influx from the thecal vasculature, leading to expansion of the antral cavity [5,26,27]. Aquaporins, transmembrane proteins, facilitate water entry and complement this osmotic mechanism [24]. Oxidative stress is a recognized cause of granulosa cell dysfunction, compromising the cellular activity required for normal follicular development [28]. Although this was not directly measured, it is possible that the antioxidant protection conferred by thymol may have preserved the metabolic activity of granulosa cells, thereby contributing to antrum formation. This is indirectly supported by the higher calcein-AM fluorescence intensity and the preserved granulosa cell ultrastructure observed in thymol-treated follicles in the present study. Nonetheless, direct functional assays of granulosa cell metabolic activity would be required to confirm this mechanism. This possibility is corroborated by a study using the same experimental model, in which an antioxidant compound added to the culture medium, *Aloe vera* extract, similarly increased the rate of antrum formation in cultured bovine secondary follicles [29].

The higher calcein-AM fluorescence intensity observed in follicles cultured with 10 µg/mL thymol reflects greater cell viability and membrane integrity over the course of the culture. Calcein-AM is a pro-fluorescent substrate whose conversion to its fluorescent form depends on intracellular esterase activity, such that fluorescence intensity reflects both plasma membrane integrity and the overall metabolic status of the cells [30]. The fluorescence signal is therefore proportional to the number of active viable cells within the follicle [2,31]. The antioxidant properties of thymol may have protected granulosa and thecal cells from oxidative damage during culture, limiting structural lesions and apoptotic events, which in turn enhanced esterase activity. This cytoprotective capacity has been reported across multiple experimental systems involving thymol [11,32,33]. Badr et al. [34] recently showed that thymol preserved spermatogenesis and restored sperm quality in rats subjected to cisplatin-induced testicular toxicity, an effect associated with reduced reactive oxygen species accumulation. Corroborating these functional findings, granulosa cells from follicles cultured in the presence of thymol generally exhibited preserved ultrastructure, consistent with the features previously reported for granulosa cells of cultured bovine secondary follicles, suggesting that the presence of this molecule has no deleterious effects, but rather may act as a protective agent during follicular culture [2,29].

At day 2 of culture, MDA levels, a well-established marker of lipid peroxidation and membrane oxidative damage [35], were higher in follicles cultured with 100 µg/mL thymol than in the control medium alone or with 10 µg/mL thymol. Additionally, follicles cultured with 1 and 10 µg/mL thymol had increased levels of nitrite after two days of culture. Nitrite is an indirect marker of nitric oxide (NO) production due to the rapid degradation of this molecule [36,37,38], and its concentration in the medium reflects the net balance between production and consumption. The NO has been associated with follicular growth, antrum formation, and viability in preantral follicles [39,40,41], and at low concentrations it acts as a signaling molecule, whereas excessive levels can be detrimental. The higher nitrite levels observed in this study could therefore be compatible with a physiological role of NO; however, NO synthase expression or activity, as well as NO bioavailability, and the signaling pathways involved were not investigated.

Thymol increased CAT and GPX1 mRNA levels, whereas SOD, PRDX6, PCNA, and GDF-9 expression did not differ between groups, indicating a selective effect on specific antioxidant transcripts rather than a broad transcriptional response. It should be noted that mRNA levels do not necessarily reflect protein abundance or activity, and post-transcriptional or translational regulation of SOD, PRDX6, and GDF-9 cannot be ruled out; protein-level analysis of these targets should be addressed in future studies. A similar selective pattern was reported by Vasconcelos et al. [1], in which eugenol increased GPX1 mRNA in bovine secondary follicles without affecting CAT or PRDX6. Unlike eugenol, thymol also increased CAT transcripts, indicating a comparatively broader effect on the antioxidant transcriptional profile. Vasconcelos et al. [42] also reported that *Ocimum gratissimum* essential oil, rich in eugenol and 1,8-cineole, increased CAT mRNA in bovine secondary follicles without altering follicular growth or morphology. Together, these findings indicate that phenolic and terpenoid compounds selectively modulate specific antioxidant transcripts in this follicular model rather than activating the entire oxidative defense gene network. Although the signaling pathways were not investigated in the present study, the upregulation of CAT and GPX1 may be related to the JAK1/STAT3 pathway, as thymol interacted directly with JAK1 in human granulosa cells under oxidative stress, restoring SOD, CAT, and GPX activities [43]. Thymol has also been reported to activate the Keap1/Nrf2/HO-1 pathway, which induces the expression of antioxidant defense genes [34]. The involvement of these pathways in bovine follicles remains speculative and requires further investigation.

## 4. Material and Methods

Unless otherwise specified, all chemicals, including thymol and culture media, were obtained from Sigma-Aldrich (St. Louis, MO, USA).

### 4.1. Ovaries

Ovaries from 60 cows were collected at a local slaughterhouse, washed with 70% ethanol, rinsed twice in TCM-199 with 100 IU penicillin and 0.1 mg/mL streptomycin, and transported to the laboratory in the same medium at 4 °C within one hour.

### 4.2. Isolation and Culture of Secondary Follicles

The secondary follicles were isolated mechanically using a 25-gauge (G) syringe, following the protocol established by Nascimento et al. [2]. The follicles were individually cultured in 100 μL microdrops of TCM-199 medium (pH 7.2–7.4) supplemented with 4 IU/mL equine chorionic gonadotropin (FOLLIGON^®^, MSD Animal Health), 10 μg/mL insulin, 5 μg/mL transferrin, 5 ng/mL selenium, 50 μg/mL ascorbic acid, 3.0 mg/mL bovine serum albumin, 2 mM glutamine, 2 mM hypoxanthine, 100 IU/mL penicillin and 0.1 mg/mL streptomycin, which was designated as TCM-199^+^. For the treatments, the follicles were in TCM-199^+^ alone or with 1, 10, or 100 μg/mL thymol (concentrations selected according to the results reported by Gunes-Bayir et al. [44]). The follicles were cultured in an incubator at 38.5 °C under 5% CO_2_ for 18 days. Every two days, 60 μL of the culture medium was replaced with an equal volume of fresh medium. At the end of the culture period, follicular diameters and the presence of an antrum were evaluated under a stereomicroscope (SMZ 645, Nikon, Tokyo, Japan). The experimental procedure was repeated twelve times, resulting in the isolation and culture of 413 follicles in total.

Follicles were examined on days 0 and 18 under an inverted microscope (Nikon Eclipse TS100, Nikon, Tokyo, Japan). At the end of culture, measurements were obtained from two perpendicular axes at the outer margin of the thecal cell layer, and the mean value was used as the final diameter (μm). The presence of a clear cavity within the granulosa cell layers indicated antrum formation.

### 4.3. Assessment of Secondary Follicle Viability and Ultrastructure

After the culture period, the follicles (n = 30 per treatment) were incubated in 100 μL drops of TCM-199 containing 4 mM calcein-AM and 2 mM ethidium homodimer-1 as described previously [30]. Fluorescence intensity was quantified using ImageJ software version 1.54 (National Institutes of Health, Bethesda, MD, USA). Ultrastructural analysis was performed in secondary follicles cultured for 18 days (n = 8 per experimental group) as previously described by Azevedo et al. [29]. Micrographs were selected to illustrate representative ultrastructural features.

### 4.4. Expression of mRNA for Antioxidant Enzymes, Cell Proliferation Marker, and Oocyte-Derived Growth Factor

Each biological replicate consisted of pooled RNA from 8 follicles (n = 8 replicates per treatment; 64 follicles per treatment in total). Based on the results of growth and viability, total RNA was extracted from samples of follicles cultured in TCM199^+^ alone or with 10 μg/mL thymol using Trizol^®^ reagent (Invitrogen, São Paulo, Brazil). After total RNA extraction, residual genomic DNA was removed by on-column DNase digestion with the PureLink™ DNase kit (Thermo Fisher Scientific, Waltham, MA, USA), according to the manufacturer’s instructions. The RNA concentration and purity were assessed by spectrophotometric analysis on a BioDrop instrument (Cambridge, UK). Complementary DNA synthesis was conducted using 1.6 μg of total RNA in a final reaction volume of 30 µL, with the superscript III first-strand synthesis kit (Invitrogen, Carlsbad, CA, USA) and random primers. The resulting cDNA was kept at −20 °C. All qPCR reactions were conducted with SYBR Green Master Mix (Applied Biosystems, Waltham, MA, USA) in a real-time PCR System (StepOnePlus, Applied Biosystems, Waltham, MA, USA). The thermal cycling program consisted of an initial denaturation and enzyme activation step at 95 °C for 10 min, followed by 40 cycles comprising 95 °C for 15 s, 58 °C for 30 s, and 72 °C for 30 s, with a final extension at 72 °C for 10 min.

Transcript levels were evaluated for genes associated with antioxidant defense, including SOD, CAT, PRDX6, and GPX1, as well as those related to cell proliferation and oocyte-derived signaling, namely PCNA and GDF-9. Glyceraldehyde-3-phosphate dehydrogenase (GAPDH) was used as the endogenous reference gene for normalization (Table 3). All samples were run in duplicate, and a no-template control was included in each assay to verify reaction specificity. The 2^−ΔΔCt^ method was used to calculate relative expression [45]. The primer sequences used are listed in Table 3.

### 4.5. Levels of Reduced Glutathione, Nitrite, and Malondialdehyde in Culture Medium

Samples of culture media were collected from each treatment after 2, 8, and 18 days to determine the levels of reduced GSH, nitrite, and MDA. Each replicate consisted of pooled medium from 8 follicles per treatment (60 µL/follicle collected on days 2 and 8, and 100 µL/follicle collected on day 18; a total of 1760 µL of pooled medium per replicate), with n = 4 independent replicates per treatment/time point. Total protein concentration was assessed using the Bradford assay, which uses Coomassie Brilliant Blue dye (Quick Start/Bradford, Bio-Rad, Hercules, CA, USA). Absorbance measurements were taken spectrophotometrically at a wavelength of 595 nm. The total protein concentration in the samples was determined by constructing a standard curve using bovine serum albumin (BSA) as the reference standard (concentrations: 0, 2.5, 5, 10, 15, 25, 35, and 50 mg/mL).

To determine the levels of GSH, the culture medium samples were added to 50% trichloroacetic acid, shaken, and centrifuged at 5000 rpm for 15 min at 4 °C. Subsequently, the supernatant was collected and added to 0.4 M Tris-HCl buffer, pH 8.9, and 0.01 M DTNB. After 1 min of reaction, the reading was performed at an absorbance of 412 nm and the values were expressed in mg/protein. To each sample, 50 µL of homogenate and 50 µL of Griess reagent (1% sulfanilamide in 5% H_3_PO_4_ and 0.1% N-(1-naphthyl)ethylenediamine dihydrochloride (NED) in distilled water at a ratio of 1:1:1:1) were added. A standard curve was generated from a serial dilution of a sodium nitrite solution. Readings were obtained at A540 nm, and the values were expressed in µM of NO_2_^−^ [46].

The MDA levels were quantified by measuring thiobarbituric acid-reactive substances (TBARS) production. To the samples, 125 µL of culture medium was added to 200 µL of 35% perchloric acid. The mixture was then centrifuged at 5000 rpm for 10 min at 4 °C. The resulting supernatant was combined with 100 µL of 1.2% thiobarbituric acid and heated at 95 °C for 30 min. Absorbance was measured at 535 nm, as described by [47].

### 4.6. Statistical Analysis

Statistical analyses were performed using GraphPad Prism software (version 9.0). Data were initially assessed for normality. Subsequently, values for follicular diameter, fluorescence intensity (for calcein-AM and ethidium homodimer-1), as well as those of levels of nitrite, MDA, and GSH were analyzed using the Kruskal–Wallis test, followed by Dunn’s post hoc test for multiple comparisons. Fisher’s exact test was used to compare rates of follicular growth, survival, and antrum formation. For PCR data, an unpaired T test was used. Statistical significance was set at *p* < 0.05.

## 5. Conclusions

The presence of 10 µg/mL thymol in culture medium increased follicular antrum formation, development, viability, and mRNA expression of CAT and GPX1. Additionally, it helped to preserve the ultrastructural morphology of oocyte and granulosa cells of follicles cultured in vitro for 18 days.

## Figures and Tables

**Table 1:** Percentage of normal follicles, antrum formation, and developing follicles after 0 and 18 days of in vitro culture.

Treatments	Morphologically Normal Follicles	Antrum Formation	Growing Follicles
TCM-199^+^	100% (103/103)	21.36% (22/103) a	64.08% (66/103) A
Thymol 1 µg/mL	100% (104/104)	25.96% (27/104) ab	71.15% (74/104) AB
Thymol 10 µg/mL	100% (102/102)	37.25% (38/102) b	77.45% (79/102) B
Thymol 100 µg/mL	100% (104/104)	39.42% (41/104) b	75.00% (78/104) AB

a, b or A, B represent significant differences between treatments (*p* < 0.05).

**Table 2:** Diameters (mean ± S.E.M) of bovine secondary follicles after 0 and 18 days of in vitro culture.

Treatments	Day 0	Day 18	Growth Day 0–18 (μm)	Daily Growth Average
**TCM-199^+^**	199.7 ± 3.88 a (n = 66)	248.8 ± 5.74 b (n = 66)	49.15 ± 4.28	2.73 ± 0.23
**Thymol** **1 µg/mL**	200.2 ± 4.18 a (n = 74)	254.4 ± 7.05 b (n = 74)	53.49 ± 5.04	2.97 ± 0.28
**Thymol** **10 µg/mL**	192.5 ± 3.52 a (n = 79)	258.1 ± 7.11 b (n = 79)	65.58 ± 5.07	3.64 ± 0.28
**Thymol** **100 µg/mL**	195.8 ± 3.51 a (n = 78)	250.1 ± 5.82 b (n = 78)	54.32 ± 4.88	3.01 ± 0.27

a, b represent statistically significant differences between days 0 and 18 (*p* < 0.05).

**Table 3:** Primers utilized to amplify messenger RNA sequences.

Gene	Primer Sequence (5′ → 3′)	Sense (S) Anti-Sense (AS)	GenBank Accession n°.
GAPDH	TGTTTGTGATGGGCGTGAACCAATG	S	GI: 402744670
	GCGCGTGGACAGTGGTTCATAA	AS	
PRDX6	GCACCTCCTCTTACTTCCCGGA	S	GI: 59858298
	TGCGGCCGATGGTAGTAT	AS	
GPX1	AACGTAGCATCGCTCTGAGG	S	GI: 156602645
	GATGCCCAAACTGGTTGCAG	AS	
SOD	GTGAACAAACCTCAACGTCGC	S	GI: 31341527
	GGGTTCTCCACCACCGTTAG	AS	
CAT	AAGTTCTGCATCGCCACTCA	S	GI: 402693375
	GGGGCCCTACTGTCAGACTA	AS	
PCNA	TGCCGAGATCTCAGTCACAT	S	GI:77735938
	TATGGCAACAGCTTCCTCCT	AS	
GDF-9	GACCCCTAAATCCAACAGAA	S	GI: 51702523.1
	AGCAGATCCACTGATGGAA	AS	

## Data Availability

The data that support the findings of this study are available from the corresponding author upon reasonable request.

## References

[1] Vasconcelos E.M., Costa F.C., Azevedo V.A.N., Barroso P.A.A., Assis E.I.T., Paulino L.R.F.M., Bezerra V.S., Silva J.R.V. (2021). Eugenol influences the expression of messenger RNAs for superoxide dismutase and glutathione peroxidase 1 in bovine secondary follicles cultured in vitro. Zygote.

[2] Nascimento D.R., Azevedo V.A.N., Barroso P.A.A., Barrozo L.G., Silva B.R., Silva A.W.B., Donato M.A.M., Peixoto C.A., Silva J.R.V. (2022). Effects of N-acetylcysteine on the growth, viability, and ultrastructure of bovine secondary follicles cultured in vitro. Animals.

[3] Nascimento D.R., Barbalho E.C., Barrozo L.G., de Assis E.I.T., Costa F.C., Silva J.R.V. (2023). The mechanisms governing the transition from preantral to early antral follicles and strategies for developing efficient culture systems that promote their in vitro growth. Zygote.

[4] Mondol T., Stodola J.L., Galletto R., Burgers P.M. (2019). PCNA accelerates the rate of nucleotide incorporation by DNA polymerase δ. Nucleic Acids Res..

[5] Vasconcelos G.L., Saraiva M.V., Costa J.J., Passos M.J., Silva A.W., Rossi R.O., Portela A.M., Duarte A.B., Magalhães-Padilha D.M., Campelo C.C. (2013). Effects of growth and differentiation factor 9 and FSH on in vitro development, viability, and mRNA expression in bovine preantral follicles. Reprod. Fertil. Dev..

[6] Orisaka M., Tajima K., Tsang B.K., Kotsuji F. (2009). Oocyte-granulosa-theca cell interactions during preantral follicular development. J. Ovarian Res..

[7] Dos Santos Silva R.L., de Sousa Barberino R., de Matos M.H.T. (2023). Impact of antioxidant supplementation during in vitro culture of preantral ovarian follicles: A review. Theriogenology.

[8] Santacruz-Márquez R., Flaws J.A., Sánchez-Peña L.D.C., Hernández-Ochoa I. (2023). Exposure to zinc oxide nanoparticles increases estradiol levels and induces an antioxidant response in antral ovarian follicles in vitro. Toxics.

[9] Von Mengden L., Klamt F., Smitz J. (2020). Redox biology of human cumulus cells: Basic concepts, impact on oocyte quality, and potential clinical use. Antioxid. Redox Signal..

[10] Bezdíček J., Sekaninová J., Janků M., Makarevič A., Luhová L., Dujíčková L., Petřivalský M. (2025). Reactive oxygen and nitrogen species: Multifaceted regulators of ovarian activity. Biol. Reprod..

[11] Caetano Filho F.F., Paulino L.R.F., Bezerra V.S., Azevedo V.A.N., Barroso P.A.A., Costa F.C., Amorim G.G., Silva J.R.V. (2024). Thymol increases primordial follicle activation, protects stromal cells, collagen fibers and down-regulates expression of mRNA for superoxide dismutase 1, catalase and periredoxin 6 in cultured bovine ovarian tissues. Anim. Reprod. Sci..

[12] Feng X., Li C., Zhang H., Zhang P., Shahzad M., Du W., Zhao X. (2024). Heat-Stress Impacts on Developing Bovine Oocytes: Unraveling Epigenetic Changes, Oxidative Stress, and Developmental Resilience. Int. J. Mol. Sci..

[13] Jamali T., Kavoosi G., Safavi M., Ardestani S.K. (2018). In vitro evaluation of the apoptotic effect of OEO and thymol in 2D and 3D cell cultures and a study of their mode of interaction with DNA. Sci. Rep..

[14] Saber T.M., Arisha A.H., Abo-Elmaaty A.M., Abdelgawad F.E., Metwally M.M., Saber T., Mansour M.F. (2021). Thymol alleviates imidacloprid-induced testicular toxicity by modulating oxidative stress and the expression of genes related to steroidogenesis and apoptosis in adult male rats. Ecotoxicol. Environ. Saf..

[15] Yousefi M., Hoseini S.M., Vatnikov Y.A., Karamyan A., Kulikov E.V. (2024). Dietary thymol supplementation promotes antioxidant responses and thermal stress resistance in rainbow trout, Oncorhynchus mykiss. Animals.

[16] Kaur R., Gaba J., Kumari S., Midha R. (2024). Thymol-based dual chemosensor for the optical detection of Cu^2+^ and Pb^2+^ ions and evaluation of its toxicity against plant growth-promoting rhizobacteria. Lett. Org. Chem..

[17] Yindeetrakul S., Triwutanon S., Sangmalee A., Rukkwamsuk T. (2025). Effect of Sericin on In Vitro Maturation of Bovine Oocytes Under Oxidative Stress Induced by Hydrogen Peroxide. Preprints.

[18] Deng Y., Wang L., Wei T., Chen Y., Wu X., Guo Y., Lin H., Tang H., Liu X. (2022). Inhibition of oocyte maturation by nitric oxide synthase 1 (NOS1) in zebrafish. Gen. Comp. Endocrinol..

[19] Mangione R., Pallisco R., Bilotta G., Marroni F., Di Pietro V., Capoccia E., Lazzarino G., Tavazzi B., Lazzarino G., Bilotta P. (2023). Bilirubin concentration in follicular fluid is elevated in infertile women, correlates with reduced levels of antioxidants and increased levels of nitric oxide metabolites, and negatively affects outcome measures in in vitro fertilization. Int. J. Mol. Sci..

[20] Garcia-Ruiz C., Fernandez-Checa J.C., Kaplowitz N. (1992). Bidirectional mechanism of reduced glutathione transport across the plasma membrane in intact rat hepatocytes and membrane vesicles. J. Biol. Chem..

[21] Oestreicher J., Morgan B. (2019). Glutathione: Subcellular distribution and membrane transport. Biochem. Cell Biol..

[22] Ichihara G., Katsumata Y., Sugiura Y., Matsuoka Y., Maeda R., Endo J., Sano M. (2023). MRP1-dependent extracellular release of glutathione induces ferroptosis in cardiomyocytes following ischemia-reperfusion. Circ. Res..

[23] Morikawa R., Kyogoku H., Lee J., Miyano T. (2022). Oocyte-derived growth factors promote the development of antrum-like structures by porcine cumulus-granulosa cells in vitro. J. Reprod. Dev..

[24] Cao L., Li S., Huang S., Shi D., Li X. (2021). AQP8 participates in oestrogen-mediated buffalo follicular development by regulating apoptosis of granulosa cells. Reprod. Dom. Anim..

[25] Maucieri A.M., Townson D.H. (2021). Evidence and manipulation of O-GlcNAcylation in granulosa cells of bovine antral follicles. Biol. Reprod..

[26] Schoenfelder M., Einspanier R. (2003). Expression of hyaluronic acid synthases and their respective hyaluronan receptors in bovine cumulus-oocyte complexes during maturation. Mol. Reprod. Dev..

[27] Parkes W.S., Amargant F., Zhou L.T., Villanueva C.E., Duncan F.E., Pritchard M.T. (2021). Hyaluronan and Collagen Are Prominent Extracellular Matrix Components in Bovine and Porcine Ovaries. Genes.

[28] Liu S., Jia Y., Meng S., Luo Y., Yang Q., Pan Z. (2023). Mechanisms of and potential treatments for oxidative stress in ovarian granulosa cells: A review. Int. J. Mol. Sci..

[29] Azevedo V.A.N., Barroso P.A.A., Vasconcelos E.M., Costa F.C., Assis E.I.T., Silva B.R., Paulino L.R.M., Silva A.W.B., Donato M.M.A., Peixoto C.A. (2022). Effects of Aloe vera extract on growth, viability, ultrastructure, and mRNA expression of antioxidant enzymes in bovine secondary follicles cultured in vitro. Anim. Reprod. Sci..

[30] Calderón-Peláez M.A., Castellanos J.E., Velandia-Romero M.L. (2025). A protocol for loading Calcein-AM into extracellular vesicles from mammalian cells for clear visualization with a fluorescence microscope coupled to a deconvolution system. PLoS ONE.

[31] Hausig-Punke F., Richter F., Hoernke M., Brendel J.C., Traeger A. (2022). Tracking the Endosomal Escape: A Closer Look at Calcein and Related Reporters. Macromol. Biosci..

[32] Khan M.R., Huang C., Zhao H., Huang H., Ren L., Faiq M., Zahoor A., Afzal M., Ansari A., An J. (2021). Antioxidant activity of thymol essential oil and inhibition of polyphenol oxidase enzyme: A case study on the enzymatic browning of harvested longan fruit. Chem. Biol. Technol. Agric..

[33] Li Z., Jin X., Wu Q., Long L., Li Y., Zhang Q., Li M., Lin H., Huang Y., Zhang C. (2022). Effects of an encapsulated thymol and carvacrol mixture on performance, antioxidant capacity, immune function, and intestinal health in broilers. Ital. J. Anim. Sci..

[34] Badr A.M., Aloyouni S., Mahran Y., Henidi H., Elmongy E.I., Alsharif H.M., Soliman S. (2025). Thymol preserves spermatogenesis and androgen production in cisplatin-induced testicular toxicity by modulating ferritinophagy, oxidative stress, and the Keap1/Nrf2/HO-1 pathway. Biomolecules.

[35] Haro Girón S., Monserrat Sanz J., Ortega M.A., Garcia-Montero C., Fraile-Martínez O., Gómez-Lahoz A.M., Boaru D.L., de Leon-Oliva D., Guijarro L.G., Atienza-Perez M. (2023). Prognostic value of malondialdehyde (MDA) in the temporal progression of chronic spinal cord injury. J. Pers. Med..

[36] Förstermann U., Sessa W.C. (2012). Nitric oxide synthases: Regulation and function. Eur. Heart J..

[37] Vitale F., Cacciottola L., Yu F.S., Barretta M., Hossay C., Donnez J., Dolmans M.M. (2023). Importance of oxygen tension in the in vitro culture of human ovarian tissue. Hum. Reprod..

[38] Qiu L., Xu Q., Lin Y., Wang S., Zhang H., Liu Y., Zhao J., Zhao Y., Xi H. (2025). Optimizing in vitro maturation of oocytes with nitric oxide-loaded microbubbles. Front. Cell Dev. Biol..

[39] Bruno J.B., Lima-Verde I.B., Martins F.S., Matos M.H.T., Lopes C.A.P., Maia-Júnior J.E., Báo S.N., Nobre Junior H.V., Maia F.D., Pessoa C. (2008). Característica histológica, ultra-estrutural e produção de nitrito de folículos pré-antrais caprinos cultivados in vitro na ausência ou presença de soro. Arq. Bras. Med. Vet. Zootec..

[40] Dubey P.K., Tripathi V., Singh R.P., Sharma G.T. (2011). Influence of nitric oxide on in vitro growth, survival, steroidogenesis, and apoptosis of follicle-stimulating hormone-stimulated preantral follicles from buffalo (*Bubalus bubalis*). J. Vet. Sci..

[41] Staicu F.D., Canha-Gouveia A., Soriano-Úbeda C., Martínez-Soto J.C., Adoamnei E., Chavarro J.E., Matás C. (2021). Nitrite and nitrate levels in the follicular fluid of human oocyte donors are associated with ovarian response and embryo quality. Front. Cell Dev. Biol..

[42] Vasconcelos E.M., Costa F.C., Azevedo V.A.N., Paulino L.R.F.M., Soares M.D., Souza A.L.P., Carneiro V.A., Brito G.S., Catunda Júnior F.E.A., Silva J.R.V. (2022). Chemical composition and effects of Ocimum gratissimum essential oil (OGEO) on the expression of mRNA for antioxidant enzymes during in vitro culture of bovine ovarian secondary follicles. J. Essent. Oil Res..

[43] Deng J., Luo C., Xie C., Duan H. (2025). Thymol mitigates oxidative stress-induced ovarian aging and restores steroidogenesis via the JAK1-STAT3 pathway. Curr. Issues Mol. Biol..

[44] Gunes-Bayir A., Kocyigit A., Guler E.M., Dadak A. (2020). In vitro hormetic effect investigation of thymol on human fibroblasts and gastric adenocarcinoma cells. Molecules.

[45] Livak K.J., Schmittgen T.D. (2001). Analysis of relative gene expression data using real-time quantitative PCR and the 2^−ΔΔ*C*_T_^ method. Methods.

[46] Green L.C., Wagner D.A., Glogowski J., Skipper P.L., Wishnok J.S., Tannenbaum S.R. (1982). Analysis of nitrate, nitrite, and [15N]nitrite in biological fluids. Anal. Biochem..

[47] Cighetti G., Debiasi S., Paroni R., Allevi P. (1999). Assessment of free and total malondialdehyde in biological matrices by gas chromatography–mass spectrometry: What is required for accurate detection. Anal. Biochem..

